# Hay Fever

**Published:** 1880-12

**Authors:** 


Hay Fever.—Dr. Blackley, London, says he knows of no specific treatment for the disease, though many drugs are capable of mitigating the symptoms. It appears that carbolic acid is the most effectual.

